# NOX2 as a Biomarker of Academic Performance: Evidence from University Students during Examination

**DOI:** 10.3390/antiox13050551

**Published:** 2024-04-30

**Authors:** Cristina Nocella, Alessandra D’Amico, Roberto Cangemi, Chiara Fossati, Fabio Pigozzi, Elena Mannacio, Vittoria Cammisotto, Simona Bartimoccia, Valentina Castellani, Gianmarco Sarto, Beatrice Simeone, Erica Rocco, Giacomo Frati, Sebastiano Sciarretta, Pasquale Pignatelli, Roberto Carnevale, SMiLe Group

**Affiliations:** 1 Department of Clinical Internal, Anesthesiology and Cardiovascular Sciences, Sapienza University of Rome, 00161 Rome, Italy; vittoria.cammisotto@uniroma1.it (V.C.); pasquale.pignatelli@uniroma1.it (P.P.); 2 Department of Medical-Surgical Sciences and Biotechnologies, Sapienza University of Rome, 04100 Latina, Italy; alessandra.damico@uniroma1.it (A.D.); simona.bartimoccia@uniroma1.it (S.B.); giacomo.frati@uniroma1.it (G.F.); sebastiano.sciarretta@uniroma1.it (S.S.); 3 Department of Translational and Precision Medicine, Sapienza University of Rome, 00185 Rome, Italy; roberto.cangemi@uniroma1.it; 4 Department of Movement, Human and Health Sciences, University of Rome “Foro Italico”, 00135 Rome, Italy; chiara.fossati@uniroma4.it (C.F.); fabio.pigozzi@uniroma4.it (F.P.); elena.mannacio@uniroma4.it (E.M.); 5 Department of General and Specialistic Surgery “Paride Stefanini”, Sapienza University of Rome, 00161 Rome, Italy; valentina.castellani@uniroma1.it; 6 Cardiology Division, ICOT, University Hospital, Sapienza University of Rome, 04100 Latina, Italy; sarto.1724518@studenti.uniroma1.it (G.S.); beatrice.simeone@uniroma1.it (B.S.); ericarocco.md@gmail.com (E.R.); 7 IRCCS Neuromed, Località Camerelle, 86077 Pozzilli, Italy; 8 Faculty of Medicine and Surgery, Course E, Sapienza University of Rome, 04100 Latina, Italy; smilegrouplatina@libero.it

**Keywords:** oxidative stress, examination-related stress, endothelial function

## Abstract

Background: Cortisol levels, oxidative stress, and lower cerebral performance seem to be closely related. This study aimed to evaluate the question of whether exam stress affected oxidative stress and endothelial function parameters in the salivary samples of students. Methods: A total of 114 healthy students were recruited. All students were subjected to a 21-item DASS questionnaire to assess perceived stress. Cortisol levels, biomarkers of oxidative stress, and endothelial function were evaluated at T0, during the semester, and T1, in the morning before the exam, in saliva samples. In vitro, HUVECs were stimulated with cortisol, and oxidative stress and endothelial function parameters were evaluated. Results: At T1, cortisol levels were significantly increased compared with the levels during the semester. Moreover, exam results correlated inversely with the DASS score at T1. In addition, NOX2, H_2_O_2_ and endothelin-1 significantly increased, while NO bioavailability decreased. In vitro, HUVECs treatment with human cortisol determined the increase of oxidative stress and the decrease of endothelial function, in association with impaired eNOS phosphorylation. Conclusion: NOX2-mediated oxidative stress is a mechanism that could mediate cortisol-induced transient endothelial dysfunction during academic examination. Therefore, strategies to monitor or modulate oxidative stress could help students to reduce the impact of examination-related stress.

## 1. Introduction

Acute stress and anxiety have been reported to increase the activity of the hypothalamus–pituitary–adrenal (HPA) axis, with a subsequent rise in cortisol levels [1]. Basal cortisol levels can provide a picture of steady-state HPA axis functioning; therefore, assessing cortisol response to a psychosocial challenge can serve as a marker of potential HPA axis dysregulation. A recent study [2] measured cortisol during cognitive testing, and its results indicate that higher HPA axis responses to testing were associated with decreases in memory [2]. On the same line, another study has demonstrated that elevated cortisol is associated with poorer cognitive function [3]. These findings are consistent with the hypothesis that hypothalamic–pituitary–adrenal axis dysregulation may be a risk factor for poorer cognitive performance in older persons [3].

Exam-correlated stress induces important changes in the hormonal profile concerning significant increases in the levels of adrenaline and cortisol [4].

Moreover, in subjects exposed to stress, a process of lipoperoxidation is started, resulting in the accumulation of reactive oxygen species (ROSs) that, if acute and prolonged, leads to oxidative stress and cellular damage. Therefore, the accumulation of ROS can manifest on an intellectual, cognitive, and emotional level. With respect to cognitive function, several studies have demonstrated that ROSs act as a double-edged sword. ROSs undoubtedly exert an essential function as signaling molecules for memory formation. Indeed, there is evidence indicating that ROSs regulate neuronal functions and, specifically, synaptic plasticity, which refers to the structural and molecular modifications at synapses that influence the strength of communication between neurons, and memory formation [5]. However, ROSs have also been shown to impair the same neuronal networks necessary for memory function, as they are neurotoxic molecules that exert their detrimental effects by oxidation of macromolecules such as enzymes and cytoskeletal proteins [6]. Therefore, maintaining tight control over ROS concentration is essential for proper neuronal development and function. NADPH-oxidase 2 (NOX2) is the main producer of ROSs. The physiological production of ROSs plays a role in immunity; however, an inappropriate response can lead to oxidative stress and tissue damage [7]. The mechanism involved in ROS-related tissue damage can be attributed to endothelial dysfunction [8], which occurs when the layer of cells lining the blood vessels loses its ability to properly regulate vascular function [9].

The relationship between cortisol and oxidative stress in academic performance has been evaluated. A team of researchers conducted a study to correlate physical exercise, oxidative stress, and academic performance. Two groups of dental students, a low-exercise group and a high-exercise group, were subjected to Dental Environment Stress (DES) questionnaires and salivary samples were collected at the beginning of the semester and the week of the examinations [10]. The results indicate that both groups showed a significant increase in DES values and a significant reduction in total antioxidant capacity (TAC) in the week of the examination. However, the high-exercise group showed a significantly lower reduction in salivary TAC compared with their counterparts in the low-exercise group, even when no relevant differences in stress levels were found between the two groups. Moreover, in fifty-four volunteers, the overall cortisol level was increased at pre-examination and during the examination as compared with the post-examination time interval, while paraoxonase activity (PON-1), an antioxidant enzyme that prevents lipoproteins oxidation [11], was significantly decreased [12].

As cortisol levels, oxidative stress and lower cerebral performance seem to be closely related, signal molecules such as stress-related ROS and their counterpart, nitric oxide (NO), in salivary samples could be reliable biomarkers for monitoring examination stress in students.

Therefore, this study aims to (i) establish a possible relationship between cortisol levels and NOX2-mediated oxidative stress in students, (ii) evaluate a relationship between cortisol-mediated NOX2 activation and endothelial dysfunction, and (iii) demonstrate whether psychic stress, cortisol, oxidative stress, and endothelial dysfunction are associated with a reduction in academic performance.

## 2. Materials and Methods

### 2.1. Study Design

A total of 203 healthy students (119 female (58.6%) and 84 male (41.4%)) aged from 19 to 34 years (mean age 22.6 ± 2.7 years) underwent detailed medical history collection before the inclusion in the study. The salivary parameters were investigated during the period of their lessons and on the day of the exam. Students who met the following criteria were included in the study: absence of stressors one or more months before the study, no medication or biologically active additives intake three or more months before the study, absence of acute or chronic somatic pathology, and signed written voluntary consent to participate in the study.

Exclusion criteria for the study were as follows: exposure to a stressor less than one month before the study; taking any medication earlier than three months before the study; acute somatic pathology; chronic somatic pathology in the acute or sub/decompensation stage; infectious disease; cancers of any localization; mental disorder; alcohol or drug abuse; unwillingness of the student to participate in the study; and absence on the day of the exam.

Considering these exclusion criteria, we enrolled 114 students (see Table 1).

The research was performed in compliance with all principles and standards of biomedical ethics. The study was approved by the local ethics committee of Sapienza University of Rome (Protocol No. 18/2020).

#### Saliva Sample Collection

All saliva samples were self-collected by participants in the morning (8–9 a.m.) into a sterile specimen container, until completing roughly 5 mL of saliva. Then, samples were added with an equal amount of sterile phosphate-buffered saline (PBS 1×, 1:1) to facilitate pipetting [13], divided into aliquots, and stored at −20 °C until they were analyzed.

Saliva samples were collected at two time points: T0, during the semester, and T1, in the morning before the final examination.

We have chosen saliva as a non-invasive sampling method, easy to collect and process. Moreover, salivary biomarkers of oxidative stress reflect local and systemic conditions [14]; in fact, blood concentrations of many redox biomarkers correlate with their salivary content [15]. Lastly, a blood test before the exam would have caused stress to some students, introducing a confounding variable.

### 2.2. Perceived Stress and Sport Participation

Perceived stress was measured using 21-item Depression Anxiety and Stress Scales (DASS). The questionnaire comprises three components, with each subscale containing seven items. The final score for each subscale is calculated by summing the scores of the corresponding items. Each question is scored from zero to three. As the DASS-21 is a shortened version of the original scale (which had 42 items), the final score for each subscale should be doubled. The validity and reliability of the DASS-21 have been established, and its usefulness has been supported in both public and clinical settings [16,17]. Moreover, we investigated sport participation of included students by an interview. We included students in the “active” group if they were engaged in sports activities on a regular basis. Otherwise, we considered students as “sedentary”.

### 2.3. Salivary NOX2 Levels

NOX2 is expressed in the oral cavity’s epithelial cells [18,19], which are present in saliva samples [20]. Thus, saliva can be used to measure NOX2.

Salivary NOX2 levels were measured by an ELISA kit according to manufacturer instructions (MyBioSource, San Diego, CA, USA). The standards and samples were plated for 1 h at 37 °C onto a micro-plate in the presence of HRP-conjugate reagent. After incubation with the chromogen solutions, plates were read at 450 nm. Values are expressed as ng/mL. Both intra-assay CV (%) and inter-assay CV (%) were less than 15%.

### 2.4. Salivary HBA Levels

Salivary hydrogen peroxide (H_2_O_2_) breakdown activity (HBA) was measured with HBA assay kit (Aurogene Rome, Italy, code HPSA-50). The % of HBA was calculated according to the following formula: % of HBA = Ac − As/Ac × 100 where Ac is the absorbance of H_2_O_2_ (1.4 mg/mL), as is the absorbance in the presence of the serum sample.

### 2.5. Salivary and Cellular H_2_O_2_ Production

The hydrogen peroxide (H_2_O_2_) was measured in the salivary samples and supernatants of treated HUVECs by using a colorimetric assay, as previously described [21]. A standard curve of H_2_O_2_ (0–200 μM) was performed for each assay. Briefly, 50 μL of samples were mixed with 3,3′,5,5′ tetramethylbenzidine (50 μL, Sigma-Aldrich, St. Louis, MO, USA) in 0.42 mol/L citrate buffer, pH 3.8, and 10 μL of horseradish peroxidase (52.5 U/mL, Sigma-Aldrich, St. Louis, MO, USA). The samples were incubated at room temperature for 20 min, and the reaction was stopped by the addition of 10 μL 18 N sulfuric acid. The reaction product was measured spectrophotometrically at 450 nm and expressed as μM.

### 2.6. Salivary and Cellular Nitric Oxide (NO) Assay

NO production was evaluated in the salivary samples and supernatants of treated HUVECs. A colorimetric assay kit (Abcam, Cambridge, UK) was used to determine the metabolites of nitric oxide (NO) (nitrites and nitrates, NOx) in 100 μL of samples under stirring conditions for 10 min at 37 °C. Values are expressed as µM. Intra-assay and inter-assay coefficients of variation were 2.9% and 1.7%, respectively.

### 2.7. Salivary Cortisol Levels

Quantitative determination of cortisol was performed by a commercial ELISA kit (Arbor Assay, Ann Arbor, MI, USA). Briefly, cortisol standard and samples were plated into a microtiter plate coated with goat anti-mouse IgG antibody in the presence of cortisol peroxidase conjugate and the cortisol monoclonal mouse antibody. The mixture was incubated for 1 h, shaking at room temperature and added with the TMB substrate. The TMB substrate reacts with the bound cortisol–peroxidase conjugate generating a signal detected by a plate reader at 450 nm. Values are expressed as ng/mL. Intra- and inter-assay coefficients of variation (CVs) were both <10%.

### 2.8. Salivary and Cellular Endothelin-1 Levels

Quantitative determination of human endothelin-1 was performed by a commercial ELISA kit (Elabscience). Values are expressed as pg/mL. Intra- and inter-assay coefficients of variation (CVs) were both <10%.

### 2.9. Cell Culture and Reagents

Human umbilical vein endothelial cells (HUVECs) (Lonza, Basel, Switzerland, Cat. N. CC-C2517A) were cultured in complete EGM-2 medium (Lonza Cat. No. CC-3162) and 10% FBS and were plated at a density of 2500 cells per cm^2^. HUVECs were seeded and grown overnight to a sub-confluence level.

Before activation, cells were pre-incubated (20 min at 37 °C) with NOX2ds-tat (10 μM; Anaspec, Fremont, CA, USA), a NOX2 inhibitor. After incubation, cells were treated with cortisol (175 or 256 ng/mL) for 3 h.

Conditioned media were collected to quantify soluble biomarkers, such as H_2_O_2_, sNOX2dp, endothelin-1 and NO, and pellets were analyzed with Western blot in order to verify p-eNOS expression. The experiments were conducted on five different batches of HUVECs.

### 2.10. Protein Detection, Electrophoresis, and Western Blot Analysis

Cells were scraped using ice-cold RIPA buffer in the presence of protease and phosphatase inhibitors cocktail (10 μg/mL; Thermo Fisher Scientific, Waltham, MA, USA) and sonicated three times. Following incubation on ice for 30 min, the samples were centrifuged at 10,000× *g* for 20 min, supernatants were recovered and mixed with 2× Laemmli sample buffer and 2-mercaptoethanol (20%).

Bradford colorimetric assay was used to determine the protein concentration and equal amounts of cell proteins (30 μg/lane) were separated by SDS-PAGE (10–12% polyacrylamide gel) and then electro-transferred onto nitrocellulose membranes by Trans-Blot Turbo (Mini Nitrocellulose, Bio-Rad, Hercules, CA, USA). Incubation was performed overnight at 4 °C with the following primary antibodies: rabbit monoclonal anti-Phospho-eNOS (Ser1177) antibody (Cell Signaling, Danvers, MA, USA), polyclonal anti-eNOS antibody (Cell Signaling) and mouse monoclonal anti-actin (Santa-Cruz Biotechnologies, Dallas, TX, USA). HRP-conjugated secondary antibodies (1:3000; Bio-Rad, CA, USA) were incubated with membranes for 1 h. Blots were then detected by enhanced chemiluminescence substrate (ECL Substrates, Bio-Rad, CA, USA). Densitometric analysis was performed using Image Lab software 6.1.0. The results are expressed as arbitrary units (AU) and represented as the mean of three independent experiments.

### 2.11. Cellular sNOX2dp

NOX2 activity was measured in cell supernatants as sNOX2-dp with a previously reported ELISA method [22]. Values are expressed as pg/mL. Intra- and inter-assay coefficients of variation (CV) were 8.95% and 9.01%, respectively.

### 2.12. Statistical Analysis

Categorical variables were reported as counts and percentage, continuous variables were expressed as mean ± standard deviation (SD), or median and interquartile ranges [IQRs]. Differences between percentages were assessed by chi-square test or Fisher exact test. Student unpaired t-test and Pearson product moment correlation analysis were used for normally distributed continuous variables. Appropriate nonparametric tests (Mann–Whitney U test, Wilcoxon signed-rank test and Spearman rank correlation test (Rs)) were employed for all the other variables.

Interventional study data were analyzed by performing a MANOVA with one within-subject factor (time: baseline (T0), i.e., 2–3 months before the exam, and on day of the exam, before the test (T1)) and one or two between-subject factors (active vs. sedentary students, students that obtain results ≤ 24 or >24). As covariates, we considered the possible random differences in age, sex, body mass index (BMI), and smoking habit. Analyses were repeated after logarithmic transformation of all the not-normally distributed continuous variables.

Probability values < 0.05 were regarded as statistically significant. All analyses were performed using computer software packages (IBM SPSS Statistics 28.0.1.1).

## 3. Results

### 3.1. Stress and Cortisol Levels

One-hundred-fourteen students were enrolled in the study (50 males, 64 females, age: 22.3 ± 2.73) (Table 1).

Baseline, median results of the exam were 28 (24–30), minimum 12 and maximum 30 cum laude, in particular, 32 obtained results ≤ 24, usually perceived as “bad results”.

**Figure 1:**
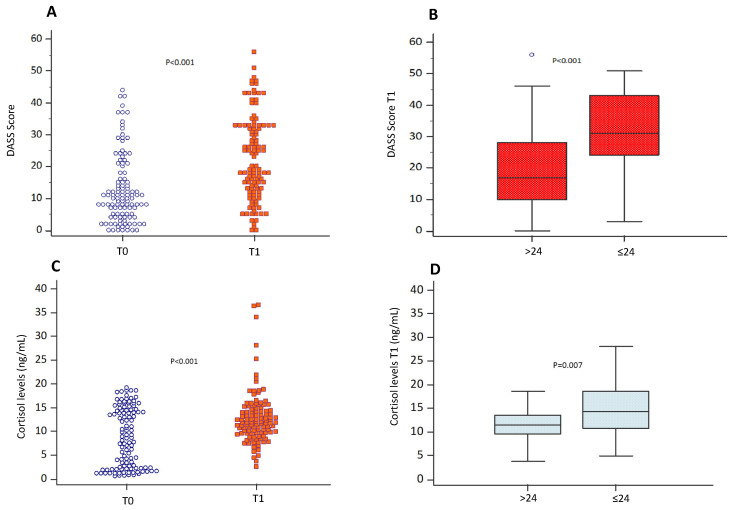
Perceived stress and cortisol levels. DASS score (**A**) and salivary cortisol levels (**C**) evaluated during the semester (T0) and on the day of the exam before the test (T1) in students (n = 144). Data are represented by dot plots and expressed as median and interquartile ranges [IQRs]. Statistical differences were assessed using the Wilcoxon signed-rank test. DASS scores (**B**) and salivary cortisol levels (**D**) on the day of the exam before the test (T1) in the group of students who obtained an exam score > 24 and ≤24, respectively. Data are expressed as median and interquartile ranges [IQRs]. Statistical differences were assessed using the Wilcoxon signed-rank test.

The baseline DASS score increased from 10 (5–18) at T0 to 19.5 (12–33) at T1 (*p* < 0.001) (Figure 1A). Participants that obtained results ≤ 24 showed higher DASS scores (31 (24–43) vs. 17 (10–28); *p* < 0.001) (Figure 1B).

Moreover, as a DASS score ≥ 15 is considered a marker of stress, 88% (28 out of the 32) of the students with a result ≤ 24 had a score indicating stress vs. 62% (51 out of 82) of the students with better results (*p* = 0.008). In all students, exam results correlated inversely with DASS score at T1 (Rs = 0.350; *p* < 0.001). No correlation was found between DASS score at T0 and the exam’s results (Rs = 0.022; *p* = 0.813).

Baseline cortisol levels increased from 8.6 (2.3–14.4) ng/mL at T0 to 11.9 (9.7–14.6) ng/mL at T1 (*p* < 0.001) (Figure 1C). At T1, cortisol levels were higher in participants with results ≤ 24 (14.3 (10.7–18.6) vs. 11.5 (9.6–13.5) ng/mL; *p* = 0.007), compared with students with better results (Figure 1D).

### 3.2. Oxidative Stress Evaluation

**Figure 2:**
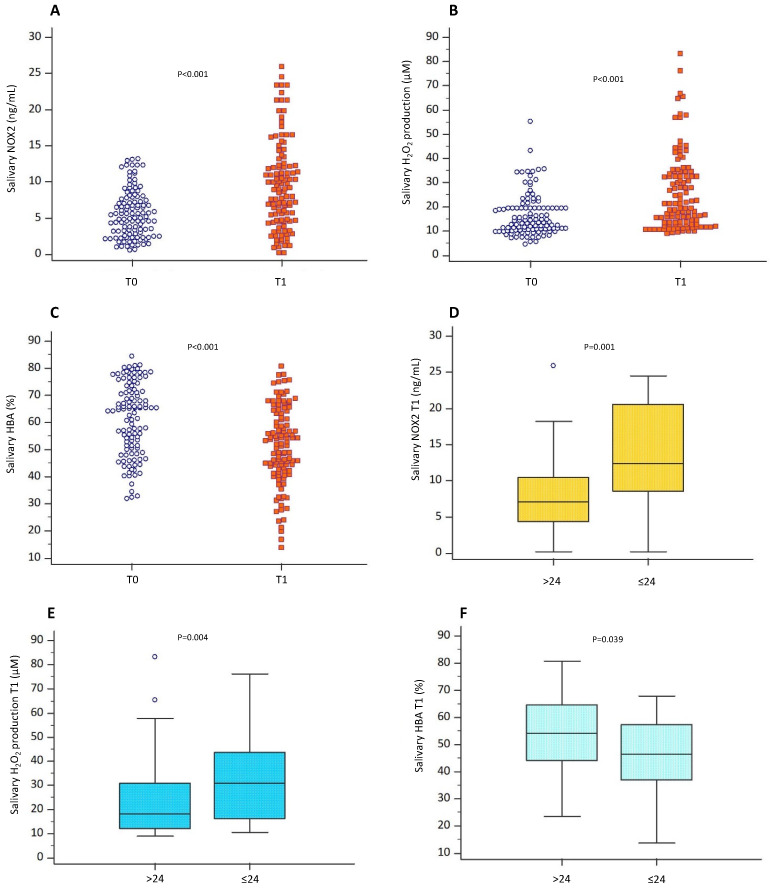
Oxidative stress evaluation. Oxidative stress levels were assessed through the evaluation of salivary levels of NOX2, (**A**) H_2_O_2_ (**B**), and HBA percentage (**C**) of participants (n = 144) during the semester (T0) and on the day of the exam before the test (T1). Data are represented by dot plots and expressed as median and interquartile ranges [IQRs]. Statistical differences were assessed using the Wilcoxon signed-rank test. Salivary levels of NOX2 (**D**), H_2_O_2_ (**E**) and HBA percentage (**F**) measured on the day the exam before the test (T1) in the group of students who obtained exam scores > 24 and ≤24, respectively. Data are expressed as median and IQRs. Statistical differences were assessed using the Wilcoxon signed-rank test.

NOX2 and H_2_O_2_ levels increased at T1 (from 5.29 (2.83–7.79) ng/mL to 8.93 (4.58–12.30) ng/mL; *p* < 0.001, and 13.5 (10.5–19.5) µM to 20.4 (12.7–33.5) µM; *p* < 0.001, respectively), while HBA decreased (64.4 (50.4–71.6) % vs. 52.4 (42.5–63.2) %; *p* < 0.001) (Figure 2A–C).

**Figure 3:**
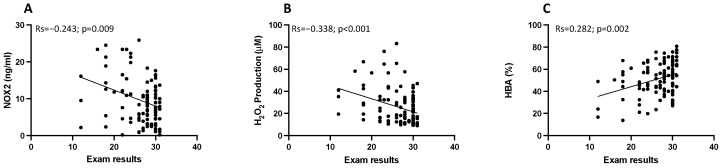
Correlation analysis examining the association between oxidative stress and exam results. Scatter plots showing significant (2-tailed) Spearman correlation of NOX2 (**A**), H_2_O_2_ production (**B**) and HBA (**C**) in vertical vs. horizontal directions of exam results. Spearman’s test was used for correlation analysis. *p* < 0.05 was considered significant.

Participants that obtained results ≤ 24 showed higher NOX2 and H_2_O_2_ than those with results > 24 (12.4 (8.5–20.5) ng/mL vs. 23.7 [4.4–10.4] ng/mL; *p* = 0.001, and 30.9 (16.3–43.7) µM vs. 18 (12–30.9) µM; *p* = 0.004) and lower HBA levels (*p* = 0.039) (Figure 2D–F). Moreover, an inverse correlation between exam results and NOX2 (Rs = −0.243; *p* = 0.009), H_2_O_2_ (Rs = −0.338; *p* < 0.001) and a positive correlation between exam results and HBA (Rs = 0.282; *p* = 0.002) were found (Figure 3A–C). Conversely, no correlation between votes and these biomarkers existed before exams.

### 3.3. Endothelial Function

**Figure 4:**
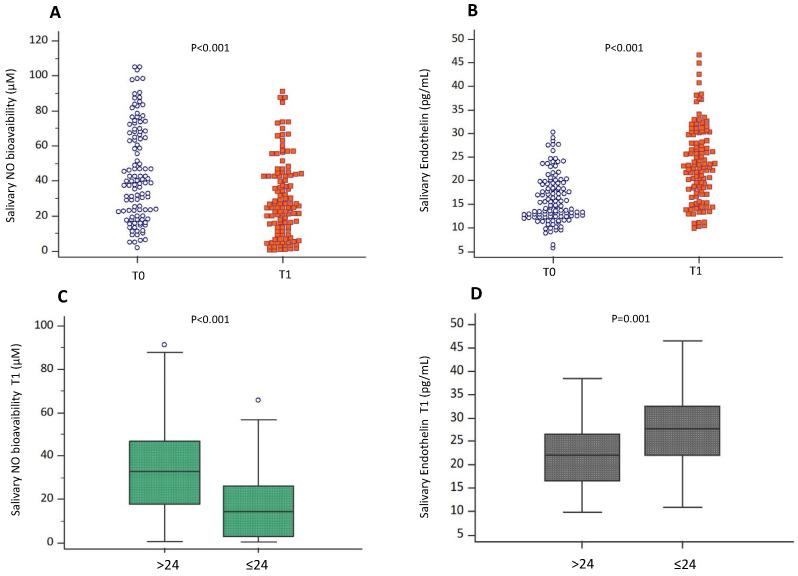
Endothelial function evaluation. Endothelial function assessed by salivary NO bioavailability (**A**) and salivary endothelin-1 levels (**B**) evaluated during the semester (T0) and on the day the exam before the test (T1) in the participants (n = 144). Data are represented by dot plots and expressed as median and interquartile ranges [IQRs]. Statistical differences were assessed using the Wilcoxon signed-rank test. Salivary NO bioavailability (**C**) and salivary endothelin-1 levels (**D**) were measured on the day of the exam before the test (T1) in the group of students who obtained respective exam scores > 24 and ≤24. Data are expressed as median and IQRs. Statistical differences were assessed using the Wilcoxon signed-rank test.

Compared with baseline, NO decreased and endothelin-1 increased at T1 (40 (22.8–68) µM vs. 25.1 (12.3–43.8) µM; *p* < 0.001, and 30.2 (12.4–19.4) pg/mL vs. 46.5 (18.1–29.7) pg/mL; *p* < 0.001 respectively) (Figure 4A,B).

**Figure 5:**
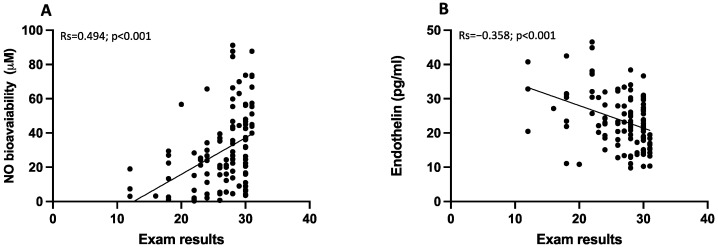
Correlation analysis examining the association between endothelial function and exam results. Scatter plots showing significant (2-tailed) Spearman correlation of NO bioavailability (**A**), and endothelin (**B**) in vertical vs horizontal directions of exam results. Spearman’s test was used for correlation analysis. *p* < 0.05 was considered significant.

Participants that obtained results ≤ 24 had lowered NO levels and increased salivary endothelin levels, respectively (*p* < 0.001, and *p* = 0.001; respectively) (Figure 4C,D). Moreover, exam results correlated positively with NO (Rs = 0.494; *p* < 0.001) and negatively with endothelin-1 (Rs = −0.358; *p* < 0.001) (Figure 5A,B).

### 3.4. Sport Participation and Academic Performance

**Figure 6:**
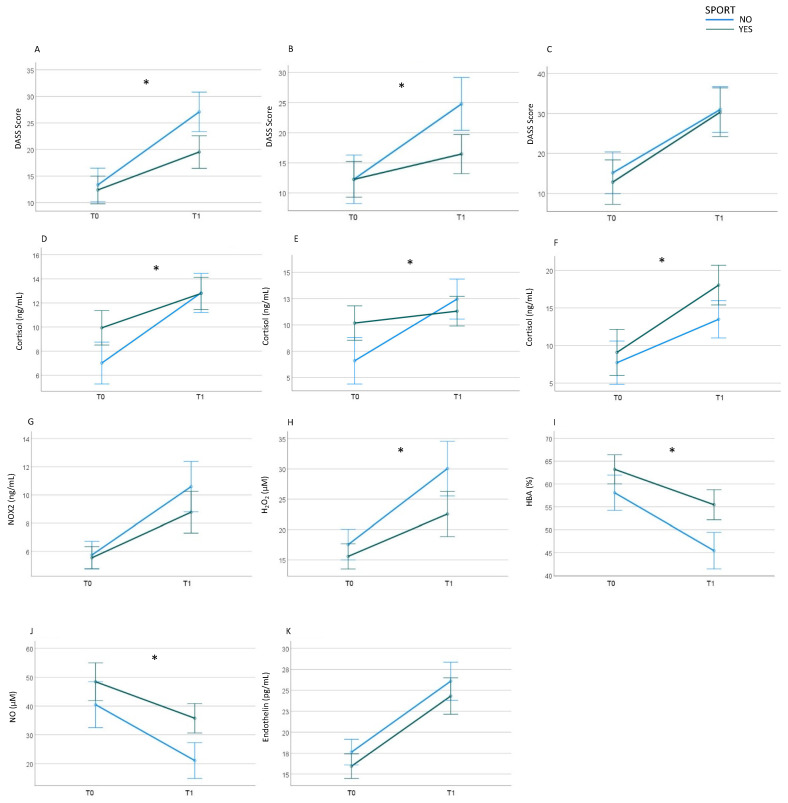
Sport participation and academic performance. DASS score (**A**) and salivary cortisol levels (**D**) in active (n = 68) (green line) and sedentary (n = 46) (blue line) participants during the semester (T0) and on the day of the exam before the test (T1). DASS score and salivary cortisol levels in active (green line) and sedentary (blue line) participants who had obtained a score higher than 24 ((**B**) and (**E**), respectively) and less than or equal to 24 ((**C**) and (**F**), respectively) during the semester (T0) and on the day the exam before the test (T1). Salivary NOX2 levels (**G**), H_2_O_2_ levels (**H**), HBA percentage (**I**), NO bioavailability (**J**), and endothelin-1 (**K**) in active (green line) and sedentary participants (blue line) at T0 and T1. * *p* < 0.05. Statistical differences were assessed using a MANOVA with one within-subject factor (time at T0 and T1) and two between-subject factors.

Active (n = 68) and sedentary (n = 46) participants exhibited comparable DASS scores before the examination (T0). However, at T1, active participants demonstrated a significantly lower increase in DASS score (F = 9.8; *p* = 0.002) (Figure 6A). Consequently, at T1, DASS scores were lower in active participants compared with their sedentary counterparts (17 (9.2–27.5) vs. 27.5 (17.5–34.2); *p* = 0.002). A repeated-measures MANOVA revealed an effect of exam results in this context (F = 5.2; *p* = 0.024): notably, students achieving exam results > 24 who engaged in sports exhibited a significantly lower increase in DASS stress scores than sedentary students with results > 24 (F = 11.7 *p* < 0.001) (Figure 6B), while the increase in DASS stress scores was comparable between active and sedentary students with exam results ≤ 24 (Figure 6C).

Baseline cortisol levels were higher in active students compared with sedentary ones (10.6 (3.7–15.5) vs. 4.2 (1.5–13.3) ng/mL; *p* = 0.009). However, the cortisol level increase at T1 was less pronounced in active students (Figure 6D), indicating an impact of sports on exam-related stress (F = 5.1; *p* = 0.025). Exam results also demonstrated an effect on this relationship (F = 8.5; *p* = 0.004); indeed, active participants exhibited a mild increase in those with favorable exam results and a substantial increase in those with exam results ≤ 24 (Figure 6E,F).

Baseline NOX2 levels did not significantly differ between active and sedentary students, a non-significant lower increase was shown among active students (*p* = 0.164) (Figure 6G), and the effect of sport was not significantly influenced by the exam’s results (*p* = 0.506).

Baseline H_2_O_2_ levels did not significantly differ between active and sedentary students. However, a lower increase was found in active students (F = 4.7; *p* = 0.032) (Figure 6H). Thus, H_2_O_2_ was lower in active participants when compared with sedentary ones (16.3 (11.4–31.9) µM vs. 27.8 (16.4–37.0) µM; *p* = 0.001). The effect of sport was not influenced by the exam’s results (*p* = 0.973).

The baseline salivary HBA was slightly elevated in active students compared with their sedentary counterparts (65.4 [54.4–76.4]% vs. 56.7 [47.5–69.1]%; *p* = 0.038), with a diminished decrease observed among active students (F = 4.7; *p* = 0.031) (Figure 6I). Consequently, a more pronounced difference was evident at T1 (54.3 (45.6–67.0)% vs. 45.8 (56.5–36.5)%; *p* < 0.001). In parallel with the oxidative stress biomarkers, the influence of sports participation remained unaffected by the examination results (*p* = 0.336).

The baseline salivary nitric oxide (NO) levels exhibited no significant difference between individuals engaged in sports activities and those who were not (*p* = 0.111). However, the reduction in salivary NO levels was less pronounced among those involved in sports (F = 7.9; *p* = 0.006). Consequently, at T1, salivary NO levels were higher in sport-participating students compared with their non-sporting counterparts (34.1 (19.8–50.4) µM vs. 16.3 (5.2–28.1) µM; *p* < 0.001) (Figure 6J). The impact of sports participation on this outcome was not significantly influenced by examination results (*p* = 0.124).

Salivary endothelin-1 levels were slightly lower in sport-involved students compared with their non-sporting counterparts, both at baseline (13.8 (12.1–18) pg/mL vs. 17 (13–20.7) pg/mL; *p* = 0.033) and at T1 (21.7 (17–26.4) pg/mL vs. 26.1 (20.4–30.8) pg/mL; *p* = 0.047). The effect of sports involvement on the increase in endothelin levels related to the examination was not statistically significant (*p* = 0.359) (Figure 6K), nor was the influence of examination results on the relationship between sports participation and examination outcomes (*p* = 0.382).

### 3.5. Effects of Cortisol on Endothelial Cell Function: In Vitro Study

Endothelial cell function was evaluated in HUVECs by measuring oxidative stress, and endothelial dysfunction biomarkers (i.e., NO bioavailability and endothelin levels) following cells stimulation with cortisol (176 or 256 ng/mL). As the concentration of cortisol in the saliva is about twenty times lower than cortisol in the serum at the same time [23], we multiplied the average exam value at T0 (8.75 ng/mL) and at T1 (12.8 ng/mL) to obtain the working concentration corresponding to the serum one.

**Figure 7:**
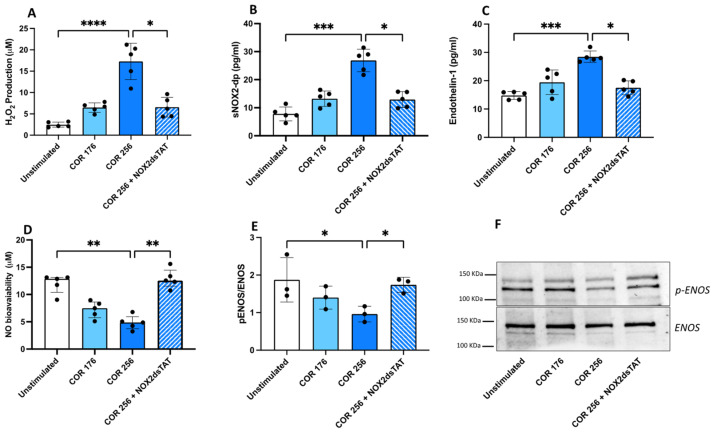
In vitro effect of cortisol on oxidative stress and endothelial function. H_2_O_2_ levels (**A**), sNOX2-dp levels (**B**), endothelin-1 levels (**C**), NO bioavailability (**D**) and p-ENOS/ENOS ratio (**E**) in HUVEC treated with different concentrations of cortisol (176 or 256 ng/mL) in the presence or not of a specific inhibitor of NOX2 (NOX2ds-tat, 10 µM). A representative image of a Western blot analysis of p-ENOS/ENOS (**F**). Data are expressed as mean ± SD; *p* values (**** *p* < 0.0001; *** *p* < 0.001; ** *p* < 0.01; * *p* < 0.05) are calculated with a non-parametric test (Kruskal–Wallis).

HUVECs stimulated with cortisol 256 ng/mL showed a significant increase of H_2_O_2_, sNOX2dp, and endothelin-1 levels, whereas NO bioavailability decreased (Figure 7A–D). Western blot analysis consistently showed a significant decrease in eNOS phosphorylation after cortisol treatment (256 ng/mL), thus regulating NO production (Figure 7E,F). To define the mechanism, HUVECs were pre-treated with NOX2ds-tat prior to their stimulation with cortisol. Results show that the inhibition of NOX2 reduces oxidative stress and endothelin-1, restores eNOS phosphorylation and increases NO bioavailability (Figure 7A–F).

## 4. Discussion

In this study, we showed that, on the day of the exam (T1), (a) cortisol levels significantly increased compared with the levels during the semester; (b) exam results correlated inversely with DASS score; (c) oxidative stress increased while endothelial function parameters decreased; (d) students engaged in sporting activities showed a significantly lower increase in DASS and cortisol levels, suggesting an impact of sport participation on exam-related stress; and (e) in vitro, HUVECs treatment with human cortisol increases oxidative stress and decreases endothelial function parameters, in association with impaired eNOS phosphorylation.

Cortisol is a hormone released by the adrenal cortex and acknowledged as “the stress hormone,” as its release is triggered by physical or psychological stress. Stress can be defined as a condition characterized by an imbalance in body functioning, an impairment in the nervous system, or emotional or physical tension [24]. Physiological and psychological stresses are implicated in the etiology of multiple pathologies, negatively affecting physical and mental health. Moreover, stress can affect cognition according to a combination of factors, such as its intensity, timing, duration, and origin [25]. A recent review has described the relationship between stress and cognition, providing evidence from both animal and human studies and confirming the significant effect of stress and stress hormones on several cognitive domains, such as global cognition, basic memory functions, and attention tasks [24].

Most of the studies exploring the relationship between stress and cortisol secretion have generally investigated the effect of long-term or chronic stress or have been conducted using laboratory-induced stressors and pharmacological approaches. Interestingly, Michaud et al., in their meta-analysis, analyzed the factors associated with the cortisol increase under natural stress conditions, such as occupational and social stressors, medical procedures, and sports-related stressors [26]. The analysis of these studies confirmed that cortisol release was associated with natural stressors, and that acute stressors had greater effect sizes than chronic stressors. Another example of a short-term and natural stress condition is the submission to an examination. Results about the association between cortisol and examination stress are discordant. Some studies have shown increased cortisol release during examination periods [27,28,29], others report unchanged cortisol levels or a reduction in cortisol release [30,31]. In this study, we have shown that, on the day of the exam, as compared with the semester period, the DASS-21 score, a well-established tool for measuring anxiety and stress [32], significantly increased along with levels of cortisol. Interestingly, we found that participants who obtained results ≤ 24, usually perceived as “bad results”, showed higher DASS scores and higher cortisol levels.

Intriguingly, we found that participants engaged in sporting activities, although they had higher baseline cortisol levels, showed a smaller increase, suggesting a protective role of sports activities on exam-related stress. The relationship between cortisol and exercise is complex, as several factors can be implicated, such as the intensity and duration of the training [33]. However, long-term exercise has been shown to increase cortisol levels, and athletes with higher cortisol levels seem to have a greater capacity for physical performance and higher motivation for competitive performances [34]. Moreover, athletes seem to respond better to stressful situations when compared with non-athletes. Indeed, they have a more efficient chronic-adaptation mechanism regarding the HPA axis [34] and the cortisol released from exercising intensely suppresses the subsequent cortisol response to psychosocial stress [35].

There is evidence that HPA activation is also linked with oxidative stress [36]. Oxidative stress is a phenomenon characterized by an imbalance between the production of reactive oxygen species (ROSs) and the activity of antioxidant defense in the prevention of the excessive accumulation of ROSs. In an experimental condition of acute psychological stress, such as the “Trier Social Stress Test” (TSST), Wiegand et al. found that, among stress-associated changes, the expression of salivary microRNA (miRNA) involved in the cellular response managing oxidative stress was higher [37]. Moreover, healthy children, after a stress situation induced by the TSST, showed increased biomarkers of lipid peroxidation compared with a control day and increased markers of antioxidant status during the stress day [38]. Consistent with these findings, we observed that sNOX2-dp, a circulating biomarker of NOX2 activation, and H_2_O_2_ levels increased on the day of the exam, with higher levels of both biomarkers significantly increased in participants that obtained results ≤ 24 when compared with those who obtained results > 24. Conversely, antioxidant capacities were significantly lower.

Oxidative stress and acute mental stress are strongly associated with endothelial dysfunction [39,40]. In the vasculature, several enzyme systems can contribute to ROS formation. Among these, NOX2 expressed in vascular cells contributes to endothelial dysfunction, as suggested by several lines of evidence. In mice, the overexpression of NOX2 induces endothelial activation [41]. In humans, deficiency of NOX2 enhanced endothelium-dependent flow-mediated vasorelaxation, and decreased markers of vascular aging and oxidative stress [42].

In addition, acute mental stresses, such as those encountered frequently during normal daily activities, affect endothelial function in healthy subjects. Endothelial dysfunction because of acute mental stress was first shown by Ghiadoni et al. In healthy subjects, without clinical vascular disease or risk factors, a mental stress test, the speech task, results in transient endothelial dysfunction as suggested by significantly reduced flow-mediated dilation (FMD) [43]. As reviewed by Poitras et al., several other studies in healthy subjects have confirmed that an acute experience of mental stress negatively affects endothelial function [44]. Here we have shown that exam-related stress decreased NO bioavailability while it increased endothelin-1, with more marked changes in participants that obtained results ≤ 24.

**Figure 8:**
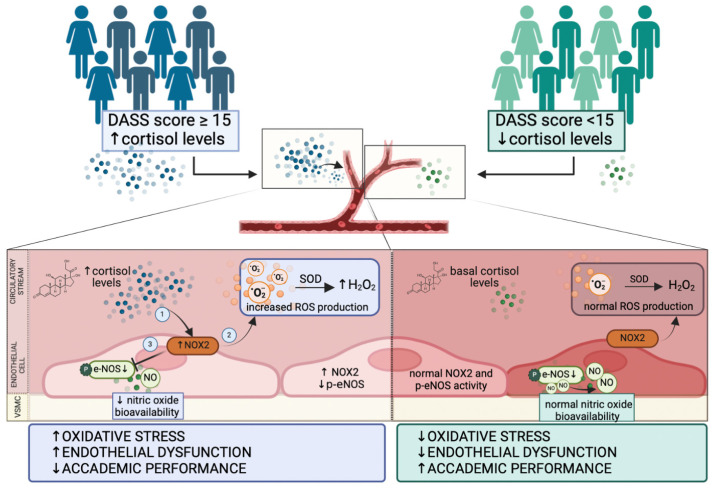
Schematic representation of cortisol-induced endothelial dysfunction and oxidative stress.

We reproduced the relationship between cortisol, oxidative stress, and endothelial function in vitro. HUVEC treated with cortisol showed increased oxidative stress and reduced NO production. Moreover, cortisol reduced the phosphorylation of eNOS, the enzyme responsible for endothelial NO production. These data suggest that, when cells are challenged with cortisol, NOX2 is activated, ROSs increase and NO is decreased because of the reduction in eNOS activation. The inhibition of NOX2 significantly attenuates the effect of cortisol on HUVEC (Figure 8).

Participants who obtained a score greater than or equal to 15 on the Depression, Anxiety and Stress Scales (DASS) test (group 1, blue) showed, simultaneously, higher concentrations of cortisol than students who achieved a final score lower than 15 (group 2, green).

High levels of cortisol in endothelial cells promote the activation of NOX2, resulting in an increased H_2_O_2_ production, which in turn can contribute to the reduction of e-NOS phosphorylation and nitric oxide release. Increased levels of oxidative stress and endothelial dysfunction can be implicated in worse academic performance.

Basal concentrations of cortisol do not affect the normal balance that exists between NOX2 and eNOS activity and which results in physiological levels of ROS and proper endothelial function.

This study has implications. Mental stress exerts a negative impact on vascular function, and oxidative stress-induced ROSs are mediators of endothelial dysfunction. Considering the high prevalence of stress today, strategies by which to protect the vascular system during periods of increased stress, such as during examinations, are necessary. For example, it has been demonstrated that the acute intake of flavanol-rich cocoa, which has a high antioxidant capacity, can be an effective dietary strategy by which to attenuate the transient impairment in endothelial function following mental stress and improve peripheral vasodilation [45].

Moreover, this study suggests that the measurement of oxidative stress levels (i.e., NOX2) could help monitor examination-related stress. Strategies to modulate oxidative stress could reduce the effect of increased cortisol levels and, therefore, moderate the impact of examination-related stress.

## 5. Limitations

This study has some limitations that warrant acknowledgment. Firstly, the evaluation of students was conducted after a written test with variable difficulties, as they originated from different courses. Additionally, being observational in nature, our study precludes the establishment of causal relationships between oxidative stress, endothelial dysfunction, and academic performance. Further, larger longitudinal and interventional studies are necessary to establish causality. Additionally, the presence of confounding variables, including rest patterns, dietary habits, sleep patterns, various stressors (e.g., personal, or financial), and the use of substances such as caffeine and alcohol, introduces potential influences on the results.

The limited sample size and sample diversity and the lack of features about race and genetic heterogeneity could also limit the generalizability of our findings.

Lastly, while the study delved into specific biochemical aspects related to stress, it did not explore crucial psychological factors such as stress resilience or coping strategies.

## 6. Conclusions

In conclusion, NOX2-mediated oxidative stress is a mechanism that could mediate cortisol-induced transient endothelial dysfunction during academic examination. Therefore, strategies by which to monitor or modulate oxidative stress could help students to reduce the impact of examination-related stress.

## Figures and Tables

**Table 1:** Anthropometric characteristics of enrolled subjects.

	Students (114)
Age (years)	22.3 ± 2.73 *
Gender (M/F)	50/64
Height (cm)	170 ± 8.66 *
Weight (kg)	64.3 ± 12.8 *
BMI	22.2 ± 3.07 *
Current smoking (n, %)	35 (30.7)
Years of smoking	3.15 ± 1.29 *
Sportive (n, %)	68 (59.6)
Years of training	10.3 ± 6.1 *

* Data are expressed as mean ± SD.

## Data Availability

The data presented in this study are available on request from the corresponding author.
